# Hepatobiliary neuroendocrine carcinoma: a case report

**DOI:** 10.1186/1752-1947-4-53

**Published:** 2010-02-18

**Authors:** Suzana Manxhuka-Kerliu, Gordana Petrusevska, Halit Maloku, Vjollca Sahatciu-Meka, Sadushe Loxha, Naim Loxha, Labinot Shahini

**Affiliations:** 1 Faculty of Medicine, Institute of Pathology, University of Prishtina, Mother Theresa St, NN, 10 000 Prishtina, Kosovo; 2 Faculty of Medicine, Institute of Pathology, St Ciril & Methodius University, Vodnjanska NN, 1000, Skopje, Former Yugoslav Republic of Macedonia; 3 Surgery Clinic, University Clinical Center of Kosovo, Mother Theresa St, NN, 10 000, Prishtina, Kosovo; 4 Faculty of Medicine, University Clinical Center of Kosovo, Mother Theresa St, NN, 10 000, Prishtina, Kosovo; 5 Faculty of Medicine, Institute of Pathology, University Clinical Center of Kosovo, Mother Theresa St. NN, 10 000, Prishtina, Kosovo; 6 Surgery Clinic, Regional Hospital of Peja, Kosovo; 7 Faculty of Medicine, Institute of Pathology, University Clinical Center of Kosovo, Mother Theresa St, NN, 10 000, Prishtina, Kosovo

## Abstract

**Introduction:**

Neuroendocrine carcinoma of the gallbladder is a rather uncommon disease. We report a case of a neuroendocrine tumor that was located in the wall of the gallbladder and that extended into the liver.

**Case presentation:**

A 52-year-old Caucasian woman presented with right-sided abdominal pain, ascites and jaundice. An MRI scan revealed a tumor mass located in the gallbladder wall and involving the liver. A partial hepatectomy and cholecystectomy were performed. Histology revealed a neuroendocrine tumor, which showed scattered Grimelius positive cells and immuno-expressed epithelial and endocrine markers. Our patient is undergoing chemotherapy treatment.

**Conclusion:**

Gastroenteropancreatic neuroendocrine tumors need a multidisciplinary approach, involving immunohistochemistry and molecular-genetic techniques.

## Introduction

Gastroenteropancreatic neuroendocrine tumors (GEP-NETs) constitute a heterogeneous group of neoplasms. Two major GEP-NET subcategories are intestinal endocrine tumors or carcinoids and pancreatic neuroendocrine tumors (PNETs).

Requests for standardization in the management of patients with gastroenteropancreatic NETs recently resulted in the development of several guidelines, including those proposed by ENETS. The TNM staging system and the grading system are based on the current WHO classifications of endocrine and digestive tumors [1-4].

The classification of GEP-NETs is based on cell mophology and the mitotic index, with well-differentiated tumors displaying monomorphic appearances and rare mitoses (<2/10 HPF), moderately-differentiated tumors displaying an intermediate morphology and mitotic rate (2-10/10 HPF) and poorly differentiated tumors consisting of pleomorphic cells with a high mitotic index (>10/10 HPF). These three histology categories of GEP-NETs (well, moderately and poorly differentiated) strongly correlates with our patient's survival. Other features of neuroendocrine tumors (such as secretion of hormones and expression of somatostatin receptors) also correlate with histological classification. "Moderately-differentiated" neuroendocrine tumors should be recognized as a subset of GET-NETs with a prognosis that is distinct from well- and poorly-differentiated tumors [5].

Most endocrine tumors are well differentiated and slow-growing. A few are poorly differentiated small-cell endocrine tumors that are rapidly growing and have a poor prognosis [6].

Even though the growth of GEP-NETs is slow in comparison with adenocarcinomas, it is generally recognized that, with the exception of 90% of insulinomas, almost all of them have long-term malignant potential. Most are malignant at the time of diagnosis, with 60% or more presenting with metastasis to the liver. The most common cause of the death is hepatic failure and malignant proliferation.

An active approach to treatment may improve our patient's quality and length of life [7].

Management strategies include surgery for cure or palliation, and a variety of other cytoreductive techniques and medical treatment, including chemotherapy and biotherapy to control symptoms due to hormone release and tumor growth, with somatostatin analogues (SSAs) and alpha-interferon. New biological agents and somatostatin-tagged radionuclides are under investigation [8].

Gallbladder neuroendocrine tumors can cause recurrent upper quadrant pain, while extrahepatic bile duct carcinoids typically produce the sudden onset of biliary colic and painless jaundice and ascites [9]. The histopathology of these tumors may reveal: carcinoids (well-differentiated endocrine tumors); small cell carcinomas (poorly differentiated endocrine carcinomas); and mixed endocrine-exocrine carcinomas [10]. Carcinoid tumors larger than 2 cm often extend into the liver and metastasize. The prognosis of small-cell carcinomas of the gallbladder is poor [11].

## Case presentation

A 52 year-old Caucasian woman presented with right-sided abdominal pain (upper quadrant pain), ascites and jaundice. She had been experiencing the abdominal pain for one year.

An MRI revealed a tumor mass located in the liver, extrahepatic bile ducts and gallbladder. Tests done at the time of admission revealed raised levels of serum amylase (490-600 IU/L), abnormal liver function (Gamma-glutamyl transpeptidase 372 IU/L; Alkaline phosphatase 1309 IU/L) and a total bilirubin of 1.90 mg/dl. With a clinical diagnosis of obstructive jaundice, our patient underwent imaging studies. The primary clinical diagnosis was liver tumor. A partial hepatectomy and cholecystectomy were performed.

Part of the liver measured 16 × 13 × 8 cm and the gallbladder 9.5 × 3.5 cm. The tumor was located in the wall of the gallbladder infiltrating the liver. The nodular mass measured 6 cm at its greatest axis, was found in the wall of the gallbladder involving the liver, and was a grey-white to yellow color. Thirteen lymph nodes diameters of 0.3 cm to 1 cm were found.

Specimens were fixed in 10% neutral buffered formalin, and paraffin embedded sections were prepared. The sections were processed for conventional histopathological examination as well as for immunohistochemistry using a standard avidin-biotin-peroxidase complex technique. Negative and positive controls were included for each batch of slides tested.

The tumor was composed of round to fusiform cells with round to ovoid hyperchromatic nuclei, arranged in sheets, nests, cords, and festoons. There were rosette-like structures and tubules present, extensive necrosis, as well as basophilic staining of the vessels. Mitotic figures were frequent.

Carcinoma cells were Grimelius positive. In addition, tumor cells immunoexpressed epithelial markers such as CK, CK7, CK19 +/-, and endocrine markers such as NSE (1+), chromogranin A (1+); while C-KIT was negative, ER negative, PR negative, Alfa fetoprotein negative, CEA negative, Ki67 positive (low <5%), Vimentin negative and synaptophysin negative.

The histopathological diagnosis was a GEP-NET tumor. Our patient is undergoing targeted therapy, including: Gleevec (Novartis) (Figure 1, Figure 2, Figure 3, Figure 4, Figure 5, Figure 6, Figure 7, Figure 8).

**Figure 1:**
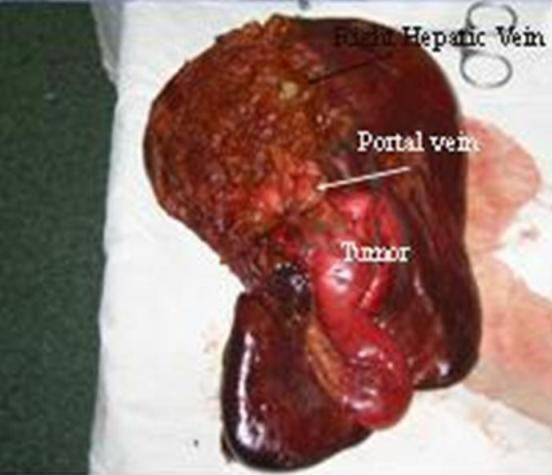
**Gross examination of the liver and gallbladder**.

**Figure 2:**
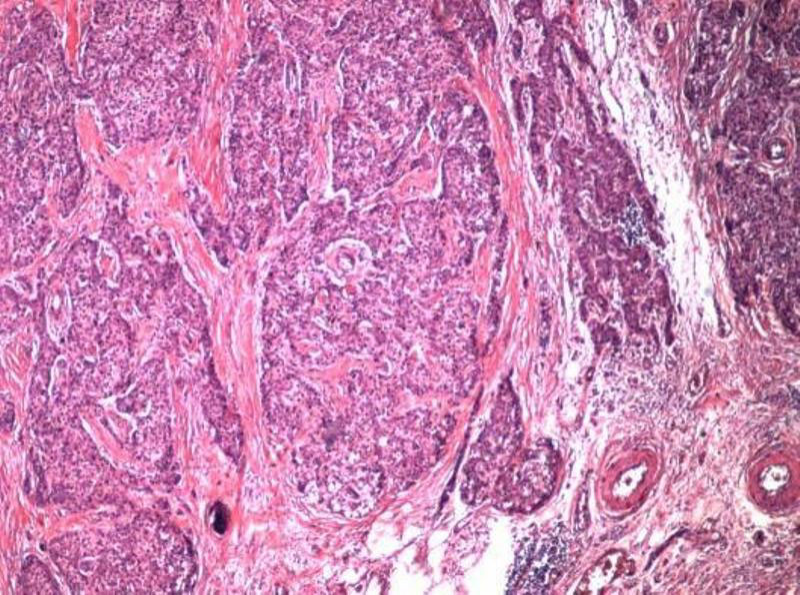
**Tumor cells invading the wall of the gallbladder Hematoxylin and eosin 5×**.

**Figure 3:**
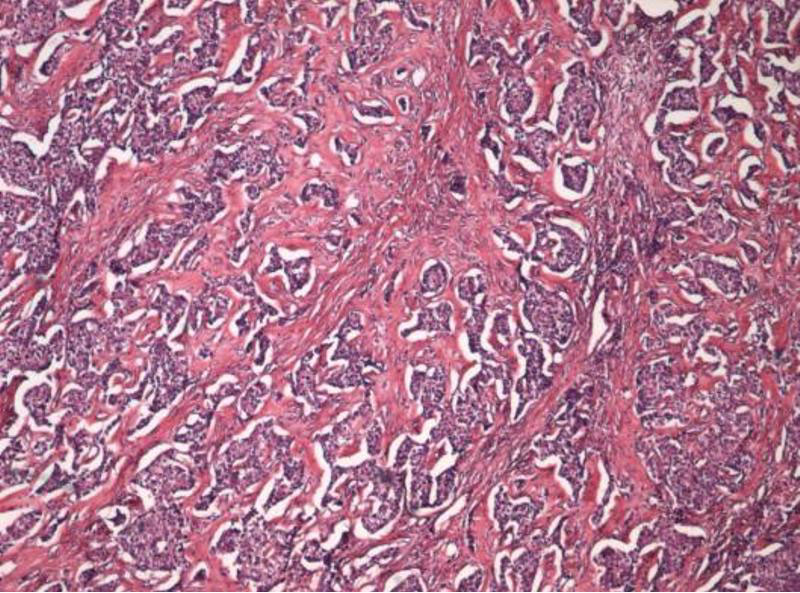
**Paraffin embedded tissue, histological examination (hematoxylin and eosin 5×)**.

**Figure 4:**
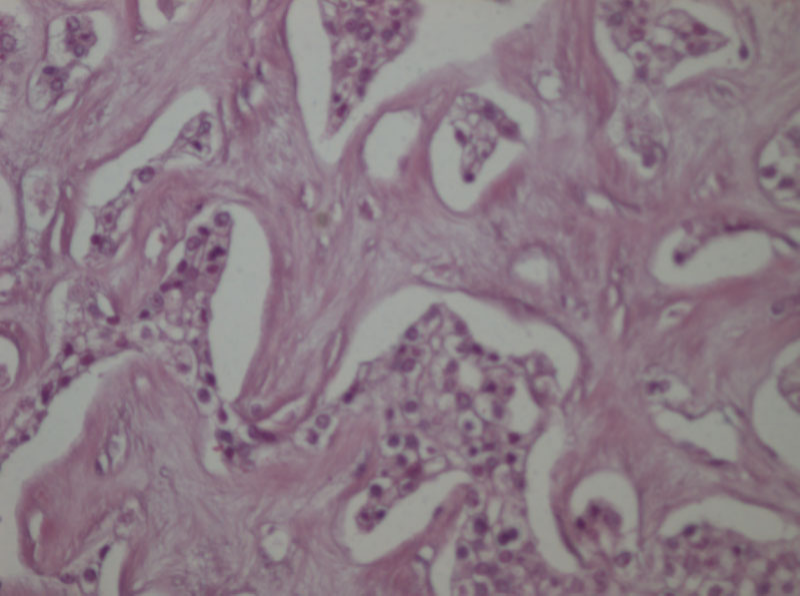
**Paraffin embedded tissue, histological examination (hematoxylin and eosin 20×)**.

**Figure 5:**
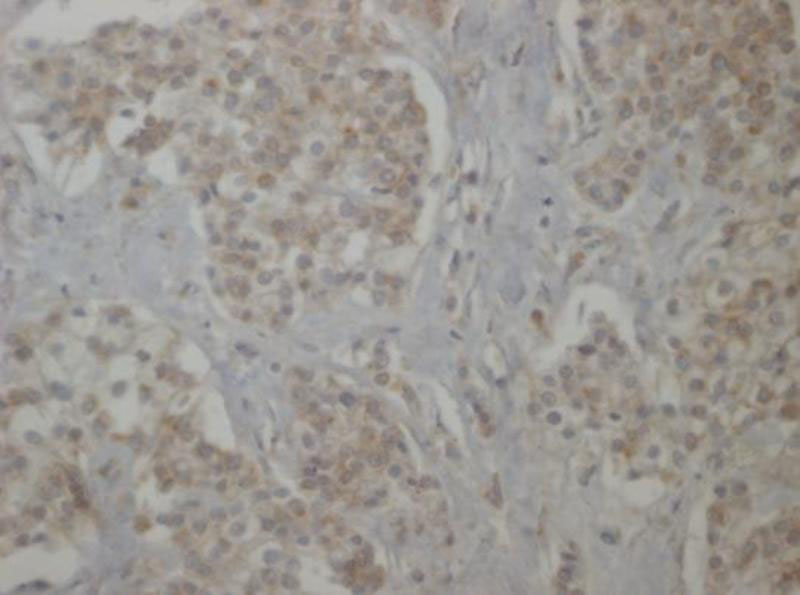
**Paraffin embedded tissue, Immunohistochemical examination, Cg A (10×)**.

**Figure 6:**
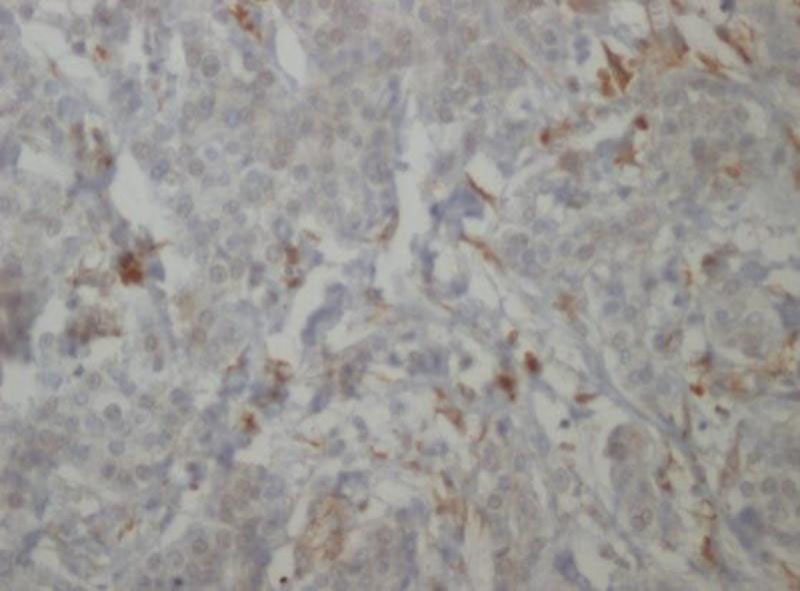
**Paraffin embedded tissue, Immunohistochemical examination, NSE (20×)**.

**Figure 7:**
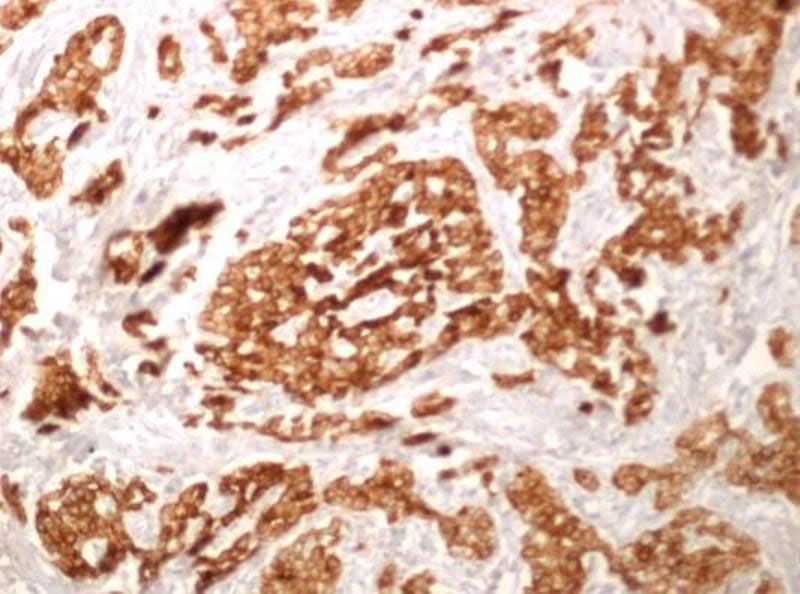
**Immunohistochemical examination, CK (20×)**.

**Figure 8:**
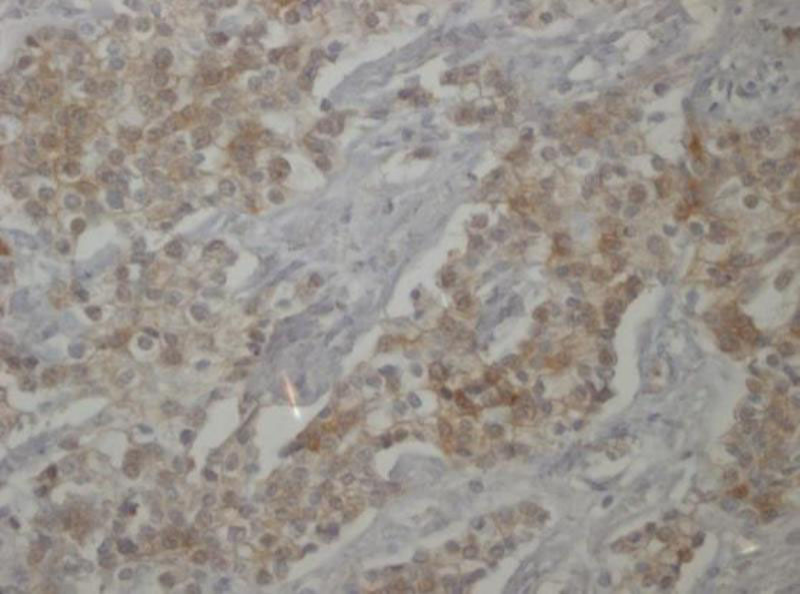
**Immunohistochemical examination, CK 19 (20×)**.

## Discussion

Hepatic neuroendocrine carcinoma is extremely rare and was first described in 1958 [12]. As of 2001, only 53 cases have been reported in English literature [9]. These tumors were mostly found in middle-age patients and were more frequently in women.

Neuroendocrine carcinoma of the gallbladder is uncommon in humans. Only 4% of epithelial tumors of the gallbladder are neuroendocrine carcinoma, which is reported to have a poor prognosis [13,14].

Bile duct and gallbladder neuroendocrine carcinomas arise from pre-existing neuroendocrine cells in the epithelium. Molecular genetic techniques will probably aid in a more clear-cut picture of the molecular background of oncogenesis and the progression of these tumors [15].

GEP-NET tumors should be treated with a multidisciplinary approach, including a partial hepatectomy, prophylactic cholecystectomy, and an excision of the lymph nodes and the primary tumor [16-19].

Receptor radionuclide therapy is a promising treatment modality for patients with neuroendocrine tumors and for whom alternative treatments are limited [20].

Since 2000, patients with somatostatin receptor-positive metastatic, inoperable GEP-NETs and malignant pheochromocytomas have been treated with the radiolabeled somatostatin analogue [^177^Lu-DOTA^0^, Tyr^3^] octreotate (^177^Lu-octreotate). Results^177 ^of Lu-octreotate treatment in these patients are promising, with a tumor size reduction in 47% of the treated patients [21].

## Conclusion

Gastroenteropancreatic neuroendocrine tumors need a multidisciplinary approach, involving immunohistochemistry and molecular-genetic techniques.

## Abbreviations

CgA: chromogranin; CK: cytokeratin; ENETS: European Neuroendocrine Tumor Society; GEP-NETs: gastroenteropancreatic neuroendocrine tumors; NSE: neuron specific enolase; PNETs: pancreatic neuroendocrine tumors; TMN: tumor-node-metastasis

## Competing interests

The authors declare that they have no competing interests.

## Authors' contributions

All authors were all involved in the conception of the case report, data collection, review of literature and writing the manuscript. SMK performed the histological examination of the gallbladder and liver and was a major contributor in writing the manuscript. GP performed the immunohistochemical examination and interpretation. HM and VSM analyzed and interpreted the clinical data. SL performed the data collection. NL performed the surgery. LSH reviewed the literature. All authors read and approved the final manuscript.

## Consent

Written informed consent was obtained from our patient for publication of this case report and accompanying images. A copy of the written consent is available for review by the Editor-in-Chief of this journal.

## References

[B1] Rindi G Bordi C Endocrine tumours of the gastrointestinal tract: aetiology, molecular pathogenesis and genetics Clin Gastroenterol 2005 19 519 534 10.1016/j.bpg.2005.03.005 16183525

[B2] Oberg K Astrup L Eriksson B Falkmer SE Falkmer UG Gustafsen J Haglund C Knigge U Vatn M Valimaki M Guidelines for the management of gastroenteropancreatic neuroendocrine tumours (including bronchopulmonary and thymic neoplasms). Part I--General overview Acta Oncol 2004 43 617 625 10.1080/02841860410018575 15545182

[B3] Rindi G Klöppel G Alhman H Caplin M Couvelard A de Herder WW Erikssson B Falchetti A Falconi M Komminoth P Körner M Lopes JM McNicol AM Nilsson O Perren A Scarpa A Scoazec JY Wiedenmann B TNM staging of foregut (neuro)endocrine tumors: a consensus proposal including a grading system Virchows Arch 2006 449 395 401 10.1007/s00428-006-0250-1 16967267 PMC1888719

[B4] Rindi G de Herder WW O'Toole D Wiedenmann B Consensus guidelines for the management of patients with digestive neuroendocrine tumors: why such guidelines and how we went about it Neuroendocrinology 2006 84 155 157 10.1159/000098006 17312374

[B5] Strosberg JR Coppola D Neumann A Kvols L Clinicopathologic analysis of well, moderately and poorly differentiated gastroenteropancreatic neuroendocrine tumors J Clin Oncol 2007 25 18S

[B6] Hamilton SR Aaktonen LA Pathology & Genetics, Tumors of the Digestive System WHO 2000 214 216

[B7] Thompson GB van Heerden JA Grant CS Carney JA Ilstrup DM Islet cell carcinomas of the pancreas: a twenty-year experience. Surgery 1988 104 1011 1017 2904180

[B8] Massironi S Sciola V Peracchi M Ciafardini C Pia Spampatti M Conte D Neuroendocrine tumors of the gastro-entero-pancreatic system World J Gastroenterol 2008 14 35 5377 5384 10.3748/wjg.14.5377 18803349 PMC2744160

[B9] Bosl GJ Yagoda A Camara LL Malignant carcinoid of the gallbladder: third reported case and review of the literature J Surg Oncol 1980 13 215 222 10.1002/jso.2930130305 6990113

[B10] Albores-Saavedra J Henson DE Tumors of the Gallbladder and Extrahepatic Bile ducts Radiographics 2002 22 387 413 11896229 10.1148/radiographics.22.2.g02mr08387

[B11] Yamamoto M Nakajo S Miyoshi N Nakai S Tahara E Endocrine cell carcinoma (Carcinoid) of the gallbladder Am J Surg Pathol 1989 13 292 302 2648878 10.1097/00000478-198904000-00004

[B12] Edmondson HA Tumors of the Liver and Intrahepatic Bile Ducts. Section 7 Fascicle 25 1958 Washington DC: Armed Forces Institute of Pathology 193 195

[B13] Iwao M Nakamura M Enjoji M Kubo H Fukotomi T Tanabe Y Nishi H Taguchi k Kotoh K Nawata H Primary hepatic carcinoid tumor: case report and review of 53 cases Med Sci Monit 2001 7 746 750 11433205

[B14] Albores-Saavedra J Molberg K Henson DE Unusual malignant epithelial tumors of the gallbladder Semin Diagn Pathol 1996 13 326 338 8946610

[B15] Maitra A Tascilar M Hruban RH Offerhaus GJ Albores-Saavedra J Small cell carcinoma of the gallbladder. A clinicopathologic, immunohistochemical, and molecular pathology study of 12 cases Am J Surg Pathol 2001 25 595 601 10.1097/00000478-200105000-00005 11342770

[B16] Kazuyoshi N Masazumi T Hideak N Hitoshi I Composite glandular-endocrine cell carcinoma of the extra-hepatic bile duct: immunohistochemical study Pathology 1993 25 90 94 10.3109/00313029309068910 8316508

[B17] Albores-Saavedra J Klimstra D Henson DE Tumor of the Gallbladder, Extrahepatic Bile Ducts, and Ampulla of Vater 2000 Washington DC: Armed Forces Institute of Pathology

[B18] Cavazzana AO Fassima AS Tollot M Ninfo V Small-cell carcinoma of the gallbladder. An immunocytochemical and ultrastructural study Pathol Res Pract 1991 187 472 476 1652129 10.1016/S0344-0338(11)80009-9

[B19] Fujii H Aotake T Horiuchi T Chiba Y Imamura Y Tanaka K Small cell carcinoma of the gallbladder: a case report and review of 53 cases in the literature Hepatogastroenterology 2001 42 1588 1593 11813580

[B20] de Keizer B van Aken MO Feelders RA de Herder WW Kam BLR van Essen M Krenning EP Kwekkeboom DJ Hormonal crises following receptor radionuclide therapy with the radiolabeled somatostatin analogue [^177^Lu-DOTA^0^, Tyr^3^] octreotate Eur J Nucl Med Mol Imaging 2008 35 4 749 755 10.1007/s00259-007-0691-z 18210106 PMC2668649

[B21] Kwekkeboom DJ Teunissen JJ Bakker WH Kooij PP de Herder WW Feelders RA van Eijck CH Esser JP Kam BL Krenning EP Radiolabeled somatostatin analog [177Lu-DOTA0,Tyr3] octreotate in patients with endocrine gastroenteropancreatic tumors J Clin Oncol 2005 23 2754 2762 10.1200/JCO.2005.08.066 15837990

